# Hospital Meetings

**Published:** 1901-03-23

**Authors:** 


### HOSPITAL MEETINGS.

### HAMPSTEAD HOSPITAL.

SIR RICHARD TEMPLE presided at the nineteenth annual meeting of the Hampstead Hospital, held at the Town Hall on Saturday last, 16th inst. The report and accounts presented showed that the ordinary income for the year was £3,632, while the legacies totalled £550. The ordinary expenditure was £2,899, and the extraordinary expenditure (repairs) £16. The deficit on the Nursing Institute accounts, now for the first time kept separately, was £42. The surplus on the year's working was thus £1,224; but from this had to be deducted the deficit of £954 at the beginning of the year, so that the balance in hand was £270. Annual subscriptions showed an increase of £146 and amounted to £877. The addition to the funds as a result of the festival dinner in May was £1,960. The number of patients treated during the year was 330, of whom 291 were discharged, 16 died, and 23 remained in the hospital at the close of the year; 4 of the deaths resulted from serious accidents, while 2 cases were hopeless when admitted. The average daily number of beds occupied was 19·7, and the average stay of each patient 23 days; 40 of the patients paid 12s. per week, and 18 paid from 2 to 5 guineas per week, the receipts from this source totalling £234—a decrease as compared with 1899 of £100. The Ladies' Association again contributed £400 to the General Fund, while an entertainment at the Conservatoire added £131, and a ball at the Grafton Galleries £95 to the Building Fund. To this fund over £9,000 has now been promised or paid. In July last some property on Hampstead Green was offered to the council, and after careful consideration accepted, and Mr. Keith D. Young, F.R.I.B.A., a resident of the borough, was appointed to erect the new building. It was calculated that an institution to hold 50 beds, with everything up to modern requirements would cost about £20,000, in addition to the cost of the site which, with expenses, would amount to not less than £6,000. A very urgent appeal for funds was therefore made. The Earl of Mansfield had consented to become a vice-president of the Hospital. The council recorded with regret the death of Mr. Wm. Anderson, one of the consulting surgeons, and of the Rev. Henry Sharpe who had been a member of the council since its institution. On November 1st, Mr. Hobbs, M.R.C.S., L.R.C.P., was appointed resident medical officer, while Mr. Gordon Strange, M.B., B.S., who had done good work as surgical and medical registrar had been appointed anæsthetist.

In moving the adoption of the report, the CHAIRMAN said they were now standing at a critical point; they were at the parting of the waters. The fountain of benevolence was opened by Dr. Strange at the base of Parliament Hill, and since then the stream had flown fairly well. But they were now approaching by the rapids to a cascade, and that they expected to surmount, after which they might fairly hope that the stream would grow rapidly broader and broader. The crux at the moment was the projected change of site. The question of where they were to build had at length been settled after considerable delay and much difficulty, but the committee were standing, and intended to stand, by the site that had been chosen. There had been some difference of opinion, even on the council itself, but it was a matter in which the majority must decide, and it behoved all to act loyally together, or there could be no concerted action and no hope of success. With reference to the site, he might mention that an endeavour was being made to effect an important improvement, and negotiations were in progress with the Metropolitan Asylums Board respecting it, and they might rest assured that it was a change which would mightily affect their future prosperity. Then, if this good site should be arranged for—and if they had their way it would be a good site—he was sure their prosperity would advance by leaps and bounds and that their friends would supply them with the necessary resources. Up till now they had been rather slack in gathering in funds, having been unsettled as to the site and so forth, but once that difficulty was surmounted and they had a first-rate site situation with every reasonable advantage that could be desired, he was sure their friends would come forward, benevolent enthusiasm would be excited, and money would flow in. He was so convinced of the moral and material benefits of the hospital that he was sure a good Providence would vouchsafe them a happy issue out of all their anxieties. He was certain that the people of Hampstead would see that of all local institutions one of the most important was a first-rate hospital. Not only was it of the greatest benefit to the sick and suffering poor, but medical science depended upon it. It was the great school, the nursery, the seminary of medical knowledge, and the most instructive cases were to be found in the hospitals. And it was also a home of training for nurses. On three counts, therefore, it was worthy of their warmest support—as a centre of benevolence, as a source of science, and as a home of training.

Mr. MALCOLM seconded the report, which was adopted unanimously.

Dr. ANDREWS then read the report of the medical staff, which, with a vote of thanks, was moved by Mr. McMillan, who mentioned that he was one of the minority on the Council with regard to the choice of the site, but accepted loyally and heartily the decision of the majority. He referred to the fact that the hospital had done specially good work as being among those that had shown the way in the matter of providing the means for those of slender purses to obtain in sickness advantages they could not get in their own homes. The motion was adopted, as was also the report of the Ladies' Association, proposed by Mr. Blyth.

On the motion of Dr. Andrews the Council were re-elected, with hearty thanks for their services, and the proposal by Dr. Strange that Mr. Malcolm be re-elected treasurer was carried with acclamation, as was also a vote of thanks to the chairman. In acknowledging this, Sir Richard paid a warm tribute to Mr. Owthwaite, the honorary secretary, and to Sir Henry Harben, to whom they looked with a lively sense of favours to come.

### ST. MARY'S HOSPITAL.

The fiftieth annual meeting of St. Mary's Hospital, Paddington, was held on Friday, 15th instant, Colonel Bannerman presiding. The report presented stated that the work during 1900 was heavier than for several years, the number relieved in the various departments being 44,117—the highest on record. Of the 281 beds in the hospital 253 were on the average constantly occupied—a very high proportion. The occasions when extra beds had to be put up were more frequent and of longer duration than in any previous year. The ordinary expenditure amounted to £27,161, the increase being due to the greater number of patients, to the higher prices of necessaries—surgical dressings and disinfectants and fuel accounting for upwards of £1,000 of the increase—and to some extent to a depreciation in the efficiency of the domestic servants. The extraordinary expenditure was £3,300, of which over £3,000 was for repairs and building. The ordinary income was £14,493, as compared with £11,766 in 1899. The extraordinary income was £9,850, of which £2,000 was from endowments and £7,850 from legacies, as compared with £12,147 in 1899. Satisfaction was expressed at the growth of income, especially that from bed endowments, which now amounted to £714 per annum. Nearly the whole of this is the product of the past eight years. Great regret was expressed at the death of Mr. Thos. Thistlethwayte, the most liberal benefactor the hospital had ever had; he and his family having been for 57 years—since before the hospital was built—foremost among its most generous and constant supporters. The inquiry system had been continued among the out-patients throughout the year. Of 20,032 cases 1,491 were selected as calling for inquiry, but investigation showed only 329, or 1·64 per cent., to be unsuitable. The number of out-patients treated was, however, higher than ever. Two further additions had been made to the nursing staff, the efficiency of which had been well maintained, though several of the sisters had been absent on active service in South Africa. The board recorded, with deep regret, the death of a probationer, Nurse Florence M. Castle. One hundred and eighty-seven patients had, on discharge, been given a three weeks' stay in the country, at the Swanley Convalescent Home, to their great benefit. The Rev. C. E. T. Whitfield had succeeded the Rev. J. Blunt Wilkinson as chaplain.

On the motion of the Chairman, seconded by Colonel Bird, the report and accounts were adopted, with a resolution of thanks to the honorary medical and surgical staff for their services, and a vote of thanks to the Chairman concluded the meeting.

### HOME HOSPITALS ASSOCIATION.

The twenty-third annual meeting of the Home Hospitals Association (for paying patients) was held at Fitzroy House on Tuesday last, 19th inst., Mr. Frederick Cox, the treasurer, presiding. The report of the Committee of Management showed that the income for the past year was £4,840, as compared with £5,269 in 1899, and the expenditure £5,223, the £383 spent in excess of revenue being carried to capital account. During the year there were 381 applications, and 304 admissions, of whom 31 were friends of patients. Of the non-admissions, 24 were for want of room, 3 cases were unsuitable, and in 3 instances the terms were beyond the patient's means. The average number of cases throughout the year was 14 and the average length of each patient's stay was 19 days. The average cost was £14 18s. 1d. Fifty-three medical practitioners in London had patients in the hospital during the year. In pursuance of Mr. Bland Sutton's suggestion at last year's annual meeting that the hospital should be rebuilt, a conjoint meeting of the committee and of the medical board was held in October. An executive committee, consisting of Mr. Bryant, Sir Henry Burdett, Mr. Cox, and Mr. Almond Hind, was appointed "to inquire into the feasibility of reconstructing or otherwise altering the present houses to meet the requirements of modern practice," but no definite plan had yet been formulated.

In moving the adoption of the report the Chairman referred to the falling-off in the number of patients, there having been 22 less than in the previous year, as accounting for the fact that they had not done so well as in the past, their income having been £429 less than in 1899, while their expenses were only £15 lower. That falling-off he had little doubt was due to the war. Many of the doctors who sent them patients had gone to the front, and many of the class from which their patients came had gone too. They had thought that some of them might have come in when they came home, for operations and so forth, but on reflection it was obvious that the reason they had not was that they went to Netley. on their return and thence to one of the numerous convalescent homes. He referred to the proposals respecting the rebuilding of the hospital, and said that the committee had met and discussed the matter, and had had the assistance of Mr. Hall, a distinguished architect, and had come to the conclusion that the suggestion to pull down and rebuild entirely on the corridor system, with operating theatres and so forth, was too expensive for them and scarcely necessary also. In the meantime they had heard that the lady next door, who had a somewhat similar business, was willing to dispose of her lease of the house and business at a moderate price. They had not, however, been able to find the ground landlord with a view to the purchase of the freehold. They were, however, rather afraid that the terms would prove prohibitive, and failing that they might turn their attention to the houses on the other side. The committee would be meeting again shortly and hoped to carry the matter a step further. They had no money, and must either beg or borrow whatever would be required. Sir Henry Burdett had expressed himself as confident that the money could be got, but whether that was too sanguine a view he could not say; and there still remained the question of whether the extended plan would pay. The Chairman concluded with a warm tribute to the Staff for the very efficient way they had carried out their duties, as they always had done.

The report was adopted, and the following were elected as the Committee of Management:—The Duke of Northumberland, the Earl of Aberdeen, Mrs. Almond Hind, Mr. W. H. Bennett, Sir Henry Burdett, Mr. Frederick Cox, Colonel H. B. Hamilton, Mr. H. S. Hammersley, Mr. T. Almond Hind, Mr. A. G. Sandeman, Sir John Watney, and two representatives of the Medical Board of Reference; and votes of thanks concluded the proceedings.

### THE FRENCH HOSPITAL.

The Annual General Meeting of the Governors of the French Hospital, Shaftesbury Avenue, was held on the 14th instant, Dr. A. Vintras presiding. The report of the committee of management was read by the Hon. Sec., M. Ernest Rüffer, and the accounts presented by the Hon. Treasurer, M. Ernest Lazarus. From these it appeared that the number of in-patients treated during the year was 875, and the out-patients 3,611. Of the 70 beds available the average number occupied was 44, each patient's stay averaging 18 days, while the cost per week was £1 9s. 10¾d. The cost of out-patients was 1s. 7d. each. The ordinary receipts for the year were £4,016 and the extraordinary —legacies and return of income tax—£1,491. The ordinary expenditure was £3,593, while £7 was spent on electric lamps. The report and accounts were adopted, and a letter to His Majesty the King was sanctioned, asking for a continuation of the patronage which, as Prince of Wales, he had hitherto accorded. Votes of thanks concluded the proceedings.

